# Gastric remnant necrosis secondary to cholesterol crystal embolization after distal gastrectomy in a gastric cancer patient: a case report

**DOI:** 10.1186/s12893-020-00716-9

**Published:** 2020-03-19

**Authors:** Jumpei Shibata, Motoi Yoshihara, Takehito Kato

**Affiliations:** Department of General Surgery, Toyohashi Municipal Hospital, 441-8570, 50 Aza Hachiken Nishi, Aotake–Cho, Toyohashi, Aichi 441-8570 Japan

**Keywords:** Gastric remnant necrosis, Cholesterol crystal embolization, Distal gastrectomy, Blue toe syndrome, Case report

## Abstract

**Background:**

Distal gastrectomy with lymph node dissection, a standard operative technique for gastric cancer treatment, is safely performed because the stomach has a rich vascular supply. Gastric remnant necrosis caused by cholesterol crystal embolization following distal gastrectomy has not been described previously. We report a case of gastric remnant necrosis in a patient with cholesterol crystal embolization.

**Case presentation:**

A 70-year-old man with a history of cholesterol crystal embolization presented to our surgery department with complaints of anorexia and dysphasia. He was diagnosed with gastric cancer invading the pyloric antrum and underwent distal gastrectomy with Billroth 2 reconstruction. On postoperative day 11, he developed abdominal pain without fever. Emergency laparotomy revealed that most parts of the remnant stomach were necrosed. Total gastrectomy with Roux-en-Y reconstruction and abscess drainage were performed. After surgery, anastomotic leakage occurred and was treated conservatively. However, the superior pancreaticoduodenal artery aneurysm suddenly ruptured and he expired.

**Conclusions:**

Gastric remnant necrosis after distal gastrectomy can be a gastrointestinal presentation of cholesterol crystal embolization. Perioperative/intraoperative risk assessments such as preventive total gastrectomy or intraoperative assessment with indocyanine green fluorescence angiography may be desirable to avoid this complication.

## Background

The stomach has a rich vascular supply provided by a collateral arterial plexus [1]. The safe performance of distal gastrectomy with lymph node dissection is due to this rich arterial network. An animal model study reported that ligation of up to 95% of the arterial supply to the stomach did not cause perfusion defects in the gastric mucosa [2]. Gastric remnant necrosis was first reported in 1953, and its mortality rate is high [3].

Cholesterol crystal embolization (CCE) is a rare manifestation of atherosclerotic disease [4]. Cholesterol crystals disseminating from atherosclerotic plaques in the aorta induce inflammation, intimal thickening, and occlusion of small arteries and arterioles [5]. They also cause exanthema, chronic renal failure, and infarctions of the brain and gastrointestinal tract [6].

Here, we report the case of a patient who developed gastric remnant necrosis after a distal gastrectomy that was caused by CCE. Our aim in presenting this report is to raise the awareness of the possible risk factors for CCE and preventive measures that may be taken to avoid this complication.

## Case presentation

**Fig. 1:**
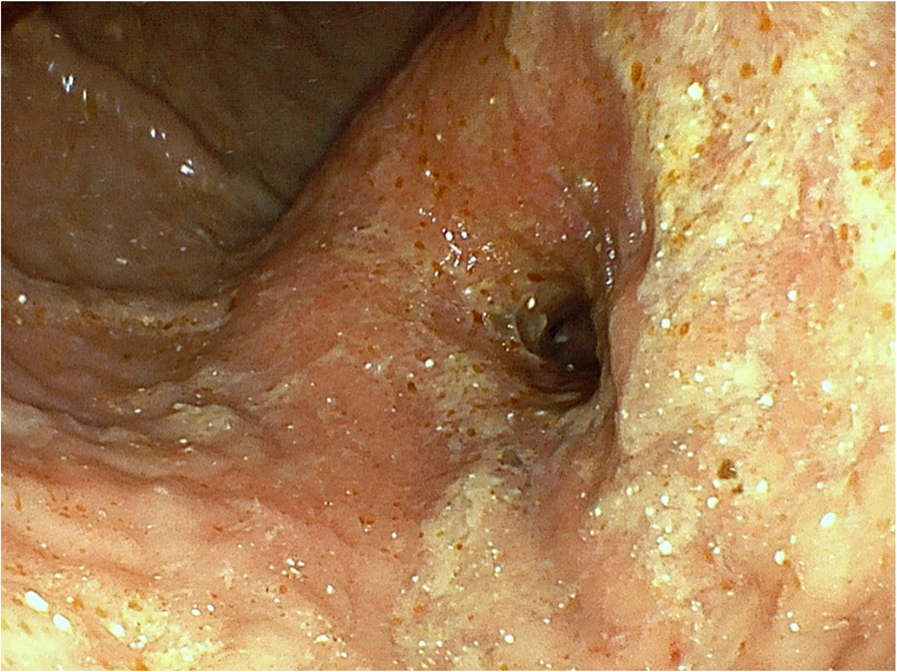
Endoscopic examination of the stomach. Endoscopy revealing a type 4 tumor circumferentially obstructing the pyloric ring

**Fig. 2:**
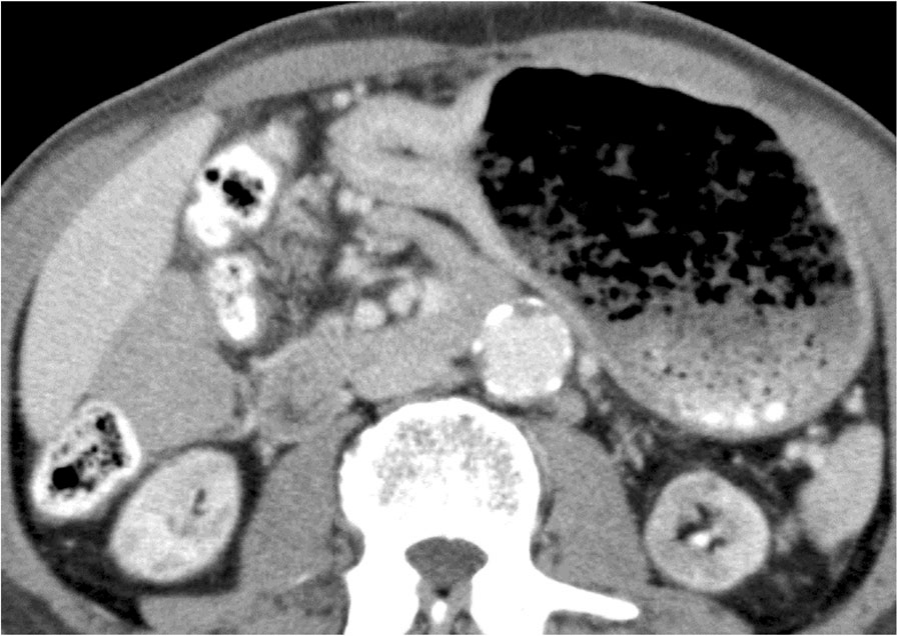
Computed tomography of the abdomen before surgery. Computed tomography of the abdomen revealing focal wall thickening of the prepyloric antrum with several enlarged perigastric lymph nodes

**Fig. 3:**
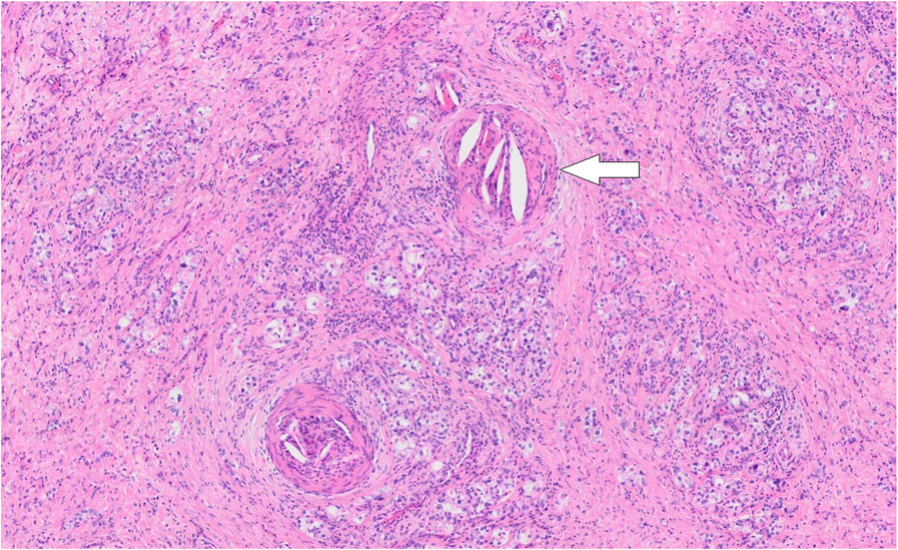
Pathological finding of the resected stomach. Multiple cholesterol emboli are found within the gastric arterial walls (arrow)

A 70-year-old man presented with anorexia and dysphagia. His past medical history was notable for type 2 diabetes, hypertension, chronic kidney disease requiring hemodialysis, and CCE. The CCE was triggered by a percutaneous coronary intervention in the context of myocardial infarction 7 years earlier. He had no history of smoking or alcohol abuse. Physical examination revealed cyanosis of both feet with dry gangrene of the distal toes but no livedo reticularis. The initial laboratory test results revealed a serum hemoglobin level of 8.0 g/dL, total white cell count of 9850/μL with 5.9% eosinophils (absolute eosinophil count, 581/μL; normal value < 500), serum carcinoembryonic antigen level of 3.2 ng/dL, and carbohydrate antigen 19–9 level of 15.3 ng/dL. Endoscopic examination revealed a type 4 tumor (Borrmann classification) circumferentially located, with invasion of the pyloric ring (Fig. 1). Biopsy revealed a poorly differentiated adenocarcinoma. Non-contrast computed tomography (CT) of the abdomen revealed focal circumferential wall thickening of the prepyloric antrum (T3) with several enlarged perigastric lymph nodes (N1) (Fig. 2) without any evidence of metastasis (M0). He underwent distal gastrectomy with Billroth 2 reconstruction. The right and left gastroepiploic arteries and right and left gastric arteries were resected, but the splenic and short gastric arteries were preserved. Lymph node dissection D2 was also performed. Postoperative histopathological findings revealed a poorly differentiated adenocarcinoma with a TNM score of pT4aN0M0. Several cholesterol emboli were found within the gastric arterial walls (Fig. 3).

On postoperative day 11, the patient developed abdominal pain without fever. Laboratory data revealed leukocytosis (white blood cell count: 17,190/μL) and an increased C-reactive protein level (21.13 mg/dL). Abdominal CT demonstrated a gastric wall discontinuity of the remnant stomach with fluid collection in the left subphrenic space. Gastric remnant necrosis was suspected, and an emergency laparotomy was performed.

**Fig. 4:**
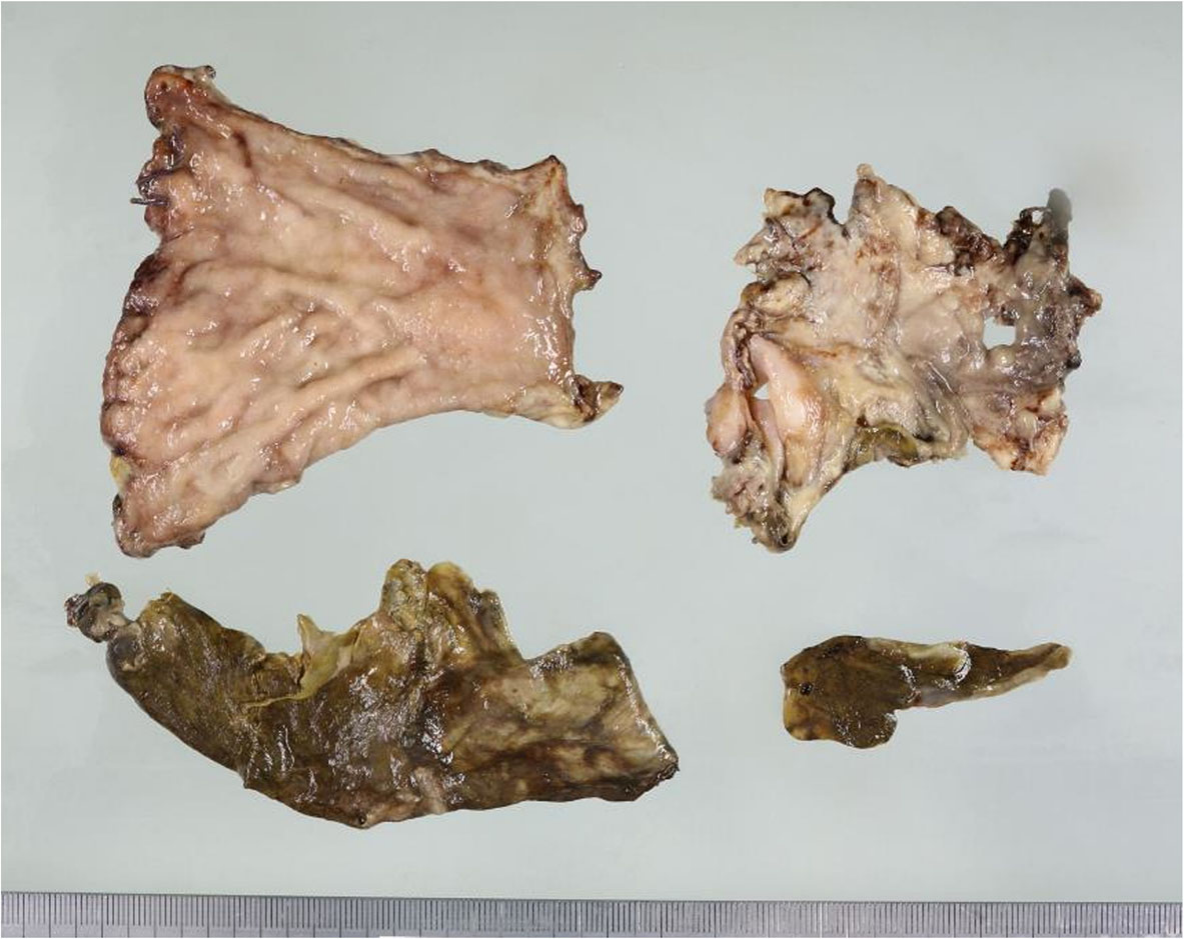
Gross findings of the gastric remnant. Most parts of the stomach, except for the greater curvature around the spleen, are completely necrosed

**Fig. 5:**
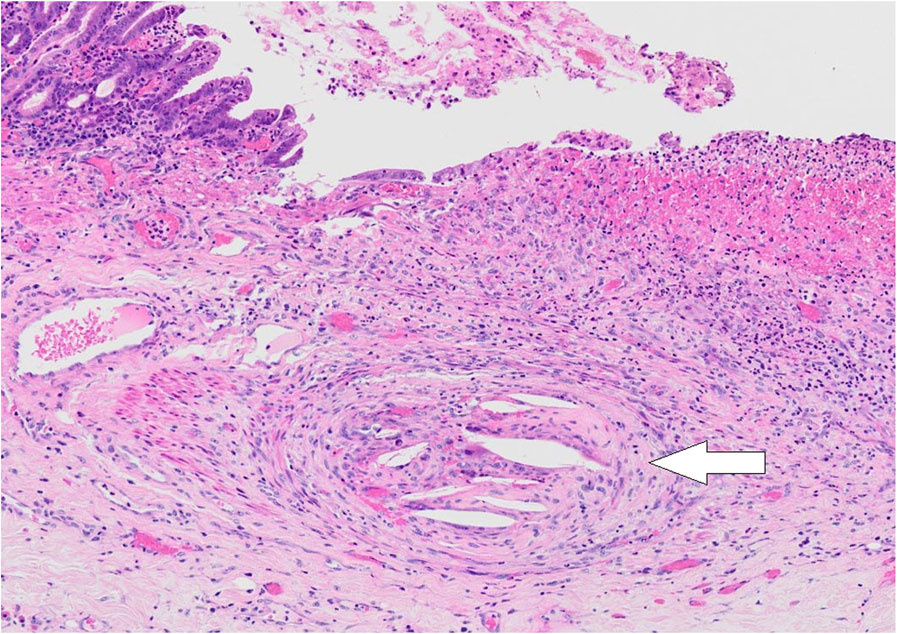
Pathological findings of the gastric remnant. The histopathological findings of the gastric remnant revealing total mucosal necrosis with cholesterol crystal emboli filling the arterioles (arrow)

Laparotomy revealed a necrotic gastric remnant. Most parts of the stomach, except for the greater curvature around the spleen, were completely necrosed (Fig. 4). Total gastrectomy with Roux-en-Y reconstruction and abscess drainage were performed. The histopathological findings of the necrotic remnant showed total mucosal necrosis with cholesterol crystal emboli filling the arterioles (Fig. 5). After surgery, anastomotic leakage occurred and was treated conservatively. However, he underwent emergency colostomy for perforation of the colon, which was due to the gastrointestinal involvement of CCE. CCE also aggravated his bilateral feet condition into amputation for gangrene. Although all of these deteriorations were treated, his superior pancreaticoduodenal artery aneurysm suddenly ruptured and he expired.

## Discussion and conclusions

We described a case where gastric remnant necrosis developed after a distal gastrectomy. To the best of our knowledge, this is the first report of gastric remnant necrosis after gastrectomy caused by CCE. This case is instructive because 1) gastric necrosis can be a presentation of CCE, in addition to kidney and skin involvement, and 2) the mortality of gastric remnant necrosis after gastrectomy is very high; therefore, it is essential to manage this risk before and during surgery.

CCE, also referred to as atheroembolism or cholesterol embolization syndrome, is a rare manifestation of atherosclerotic disease [4]. It occurs when an atherosclerotic plaque in the aorta or another major artery ruptures and releases cholesterol crystals and atheroma debris into the bloodstream, eventually occluding small arteries and arterioles, and resulting in severe end-organ damage [7]. CCE can be induced after iatrogenic mechanical trauma such as vascular surgery, angiography, or angioplasty, or as a side-effect of medications (anticoagulants or thrombolytics) targeting the coagulation system [8]. This patient developed CCE after percutaneous coronary intervention. The incidence of CCE has been reported to be 1.4% after coronary catheterization [9].

CCE affects multiple organs, especially those vulnerable to low blood perfusion such as the skin, kidneys, gastrointestinal system, and brain [10]. The gastrointestinal tract is the third-most frequent organ affected (13.4%) next to the skin (15.5%) and kidneys (31.5%) [11]. Within the gastrointestinal tract, the stomach is also the third-most common site (12.3%), next to the small intestine (33%) and colon (42.3%) [12]. Two patterns of presentation of CCE in the digestive tract are recognized: 1) acute catastrophic multiorgan disorder with poor prognosis and 2) chronic indolent gastrointestinal diseases such as abdominal pain, diarrhea, and gastrointestinal bleeding secondary to ischemic colitis [8] [13]. A catastrophic case of CCE leading to postoperative bowel infarction has been described [14]. However, no previous case of gastric necrosis after gastrectomy caused by CCE has been reported.

The stomach has a rich blood supply and an extensive submucosal plexus. Gastric necrosis in patients with CCE may not have been reported because of the stomach’s abundant blood flow. Nevertheless, in our case, transient reduction of blood supply into the gastric remnant occurred during surgery, eventually leading to a postoperative catastrophe. The mortality associated with gastric remnant necrosis has been reported to be as high as 70% [15].

The main arterial feeders of the remnant stomach after distal gastrectomy with D2 lymph node dissection are the short gastric artery, the posterior gastric artery, and the left subphrenic artery. When these arteries are preserved, blood flow to the gastric remnant is usually sufficient. However, this was not the case here. Interruption of the blood flow to the gastric remnant causes necrosis. Several reported cases have occurred secondary to splenic infarction [3]. However, some cases with a preserved splenic artery, as in the present case, have also been reported [16]. The patient in our case suffered subtotal necrosis of the remnant stomach, revealing severe blood flow reduction. The general etiology of interruption of blood flow include arteriosclerosis, atrial fibrillation, sepsis, or venous thrombosis [17]. In our patient, CCE obstructed the small arteries and arterioles within the submucosal plexus, and the resultant insufficient blood perfusion to the gastric mucosa led to necrosis of the remnant stomach.

Because the mortality of gastric remnant necrosis is very high, prevention is essential. CT angiography can be used to assess preoperative perfusion from the large vessels of the gastric walls [18]. However, given that CCE occludes the small arteries and arterioles, CT angiography might not have been revealing. It is possible that it would have been preferable to perform total gastrectomy initially. We hypothesize that arterial flow to the jejunum is preserved because there is no resected vasculature of the jejunum. In contrast, blood flow to the lower part of the esophagus is supplied by esophageal branches of the left gastric artery, left subphrenic artery, and proper esophagus arteries. To sustain sufficient esophageal blood flow, we should preserve these vessels during the total gastrectomy operation. In addition, a systematic review of the literature suggested the effectiveness of indocyanine green fluorescence angiography for intraoperative assessment of gastrointestinal anastomotic perfusion [19]. In this case, we should have assessed the risk of low blood flow of the remnant stomach; intraoperative indocyanine green fluorescence angiography would have been the preferred imaging modality. In addition, upper gastrointestinal endoscopy could be preferable to detect this rare complication early.

In conclusion, we encountered a case of gastric remnant necrosis caused by CCE after distal gastrectomy. Gastric necrosis can be a gastrointestinal presentation of CCE, and perioperative/intraoperative risk assessments may help prevent this complication.

## Data Availability

Data sharing is not applicable to this article as no datasets were generated or analysed during the current study.

## Abbreviations

CCE: Cholesterol crystal embolization
CT: Computed tomography
